# Weekly treatment with SAMiRNA targeting the androgen receptor ameliorates androgenetic alopecia

**DOI:** 10.1038/s41598-022-05544-w

**Published:** 2022-01-31

**Authors:** Sung-Il Yun, Sang-Kyu Lee, Eun-Ah Goh, Oh Seung Kwon, Woorim Choi, Jangseon Kim, Mi Sun Lee, Soon Ja Choi, Seung Sik Lim, Tae Kee Moon, Sin Hae Kim, Keeyeol Kyong, Gaewon Nam, Han-Oh Park

**Affiliations:** 1 Bioneer Corporation, 8-11 Munpyeongseo-ro, Daedeok-gu, Daejeon, 34302 Republic of Korea; 2 siRNAgen Therapeutics, Daejeon, 34302 Republic of Korea; 3 Ellead Skin Research Center, Ellead, Seongnam, 13590 Republic of Korea; 4 Department of Bio-Cosmetics, Seowon University, 377-3 Musimseoro, Seowon-gu, Cheongju, 28674 Republic of Korea

**Keywords:** Biotechnology, Drug discovery, Health care

## Abstract

Androgenetic alopecia (AGA) is the most common type of hair loss in men and women. Dihydrotestosterone (DHT) and androgen receptor (AR) levels are increased in patients with AGA, and DHT-AR signaling correlates strongly with AGA pathogenesis. In this study, treatment with self-assembled micelle inhibitory RNA (SAMiRNA) nanoparticle-type siRNA selectively suppressed AR expression in vitro. Clinical studies with application of SAMiRNA to the scalp and massaging to deliver it to the hair follicle confirmed its efficacy in AGA. For identification of a potent SAMiRNA for AR silencing, 547 SAMiRNA candidates were synthesized and screened. SAMiRNA-AR68 (AR68) was the most potent and could be efficiently delivered to human follicle dermal papilla cells (HFDPCs) and hair follicles, and this treatment decreased the AR mRNA and protein levels. We confirmed that 10 µM AR68 elicits no innate immune response in human PBMCs and no cytotoxicity up to 20 µM with HFDP and HaCaT cells. Clinical studies were performed in a randomized and double-blind manner with two different doses and frequencies. In the low-dose (0.5 mg/ml) clinical study, AR68 was applied three times per week for 24 weeks, and through quantitative analysis using a phototrichogram, we confirmed increases in total hair counts. In the 24-week long high-dose (5 mg/ml) clinical study, AR68 showed average additional hair growth of 1.3-1.9 hairs/cm^2^ per month, which is comparable to finasteride. No side effects were observed. Therefore, SAMiRNA targeting AR mRNA is a potential novel topical treatment for AGA.

## Introduction

Androgenetic alopecia (AGA), commonly known as male pattern hair loss (MPHL) in men and female pattern hair loss (FPHL) in women, is the most common type of progressive hair loss^1–3^. Androgens, male hormones, are one of the causes of AGA^4^. Although the relationship between FPHL and androgen is unclear, FPHL is accompanied by hair follicle miniaturization and hair thinning, similar to MPHL^5^. The pathogenesis of both diseases is not yet completely understood, but its incidence has increased in recent years.

Androgen and AR signaling plays an essential role in regulating the hair cycle and skin pathogenesis, including in AGA^6–8^. Endogenous androgens include testosterone and dihydrotestosterone (DHT). DHT is a more potent androgen synthesized from testosterone by 5-α reductase and exhibits an ~ tenfold higher binding affinity with the androgen receptor (AR). DHT and AR levels are elevated in patients with AGA, and it has been reported that DHT-AR signaling is closely related to AGA pathogenesis^9–13^. Finasteride and dutasteride, which were developed as 5-α reductase inhibitors, have been approved by the FDA and are being used as the main treatments for AGA. However, these drugs have several side effects, such as a decrease in libido via decreases in DHT^14,15^. Hence, there is a need for a new treatment for AGA without such side effects. Since 5-α reductase is produced in various cells, including the male and female reproductive tracts, testes, and ovaries, inhibition of DHT synthesis by local treatment is not effective, and only systemic administration will result in effective treatment. Therefore, the best way to block DHT-AR signaling without inhibition of systematic DHT synthesis is suppression of AR expression in hair tissue alone. This method constitutes an appropriate strategy for AGA treatment with minimal side effects.

AR, a nuclear receptor, is mainly expressed in dermal papilla cells (DPCs) of the human hair follicle^16–18^. When DHT enters the cell, the AR protein forms a dimer with it in the cytosol, translocates to the nucleus and acts as a transcription factor to promote the expression of inhibitory proteins of hair growth (such as TGF-β and DKK-1)^8,19–23^. Naito and colleagues reported that DHT-induced inhibition of hair regrowth did not occur in AR knockout (ARKO) mice and that ARKO mice showed an extended anagen phase in the second hair cycle compared to wild-type mice^24^. DHT-AR signaling is also involved in hair follicular miniaturization^2,4^, leading to apoptosis of DPCs^25^ and keratinocytes^26^ and the progression of AGA. Based on the essential role of AR in AGA pathogenesis, suppression of AR expression may be an ideal approach for AGA treatment.

RNA interference (RNAi) has the advantage of enabling ‘disease-modifying drugs’, as it can reduce the expression of disease-causing genes in a sequence-specific manner. Therefore, siRNA-based cosmeceuticals have been evaluated for enhanced potency^27–31^. However, the RNAi formulations developed thus far have had low efficiency due to limited delivery to skin cells and hair follicles^32–34^. Additionally, siRNA has intrinsic inflammatory side effects, which may exacerbate alopecia due to the inflammatory response in hair tissues. FDA-approved siRNA drugs (Onpattro and Givlaari) are also associated with an inflammatory response due to nonspecific innate immune stimulation. Therefore, the use of conventional siRNA-based nanoparticle technology is limited in hair-loss treatment. To overcome the hurdle of the delivery and side effects of siRNA for AGA treatment, we applied self-assembled micelle inhibitory RNA (SAMiRNA) technology.

SAMiRNA is a new type of siRNA nanoparticle that does not result in innate immune stimulation. It is a dual-conjugated DNA/RNA heteroduplex with hydrophilic and hydrophobic conjugates at each end of oligonucleotides, and thus, SAMiRNA spontaneously assembles to form nanoparticles^35–37^. SAMiRNA nanoparticles have a size of less than 100 nm and a neutral charge, which is ideal for delivery into cells^38^. Nanoparticles can be more efficiently delivered to hair follicles than small molecules through the pumping effect of massage after application^39,40^. Additionally, SAMiRNA nanoparticles do not show innate immune stimulation effects, unlike conventional siRNA formulations. Indeed, an intrinsic problem with siRNA is that it acts as a Toll-like receptor 3 (TLR3) ligand, and lipid nanoparticle (LNP) formulations cause additional stimulation by cationic lipids of LNP^33,41,42^. SAMiRNA spontaneously forms spherical nanoparticles without cationic lipids in an aqueous solution. Unlike siRNA, SAMiRNA also has no innate immune stimulation due to steric hindrance that prevents binding to TLR3 by the conjugates attached to both ends^35–37^. Therefore, SAMiRNA may be an ideal solution to overcome the above two problems of siRNA in the development of AGA treatment. Based on this theoretical background, we performed the following analyses.

In this study, 547 SAMiRNA nanoparticles containing each specific AR mRNA region were designed, synthesized, and screened. Among the SAMiRNA candidates, SAMiRNA-AR68 (AR68) had the most potent silencing efficacy, lowering the levels of AR mRNA and protein in DPCs and hair follicles. In addition, human peripheral blood mononuclear cells (PBMCs) were treated with AR68 to confirm its safety through innate immune response analysis. After confirming its efficacy and safety in vitro, we further evaluated AR68 in clinical studies. Low-dose (0.5 mg/ml) treatment applied three times per week for 24 weeks showed efficacy and safety. For more potency and convenience, a clinical study with a high-dose (5 mg/ml) formulation applied once per week was performed for 24 weeks, and the high-dose data showed efficacy similar to that of finasteride without side effects.

## Results

### Screening of potent SAMiRNA for AR silencing

**Figure 1:**
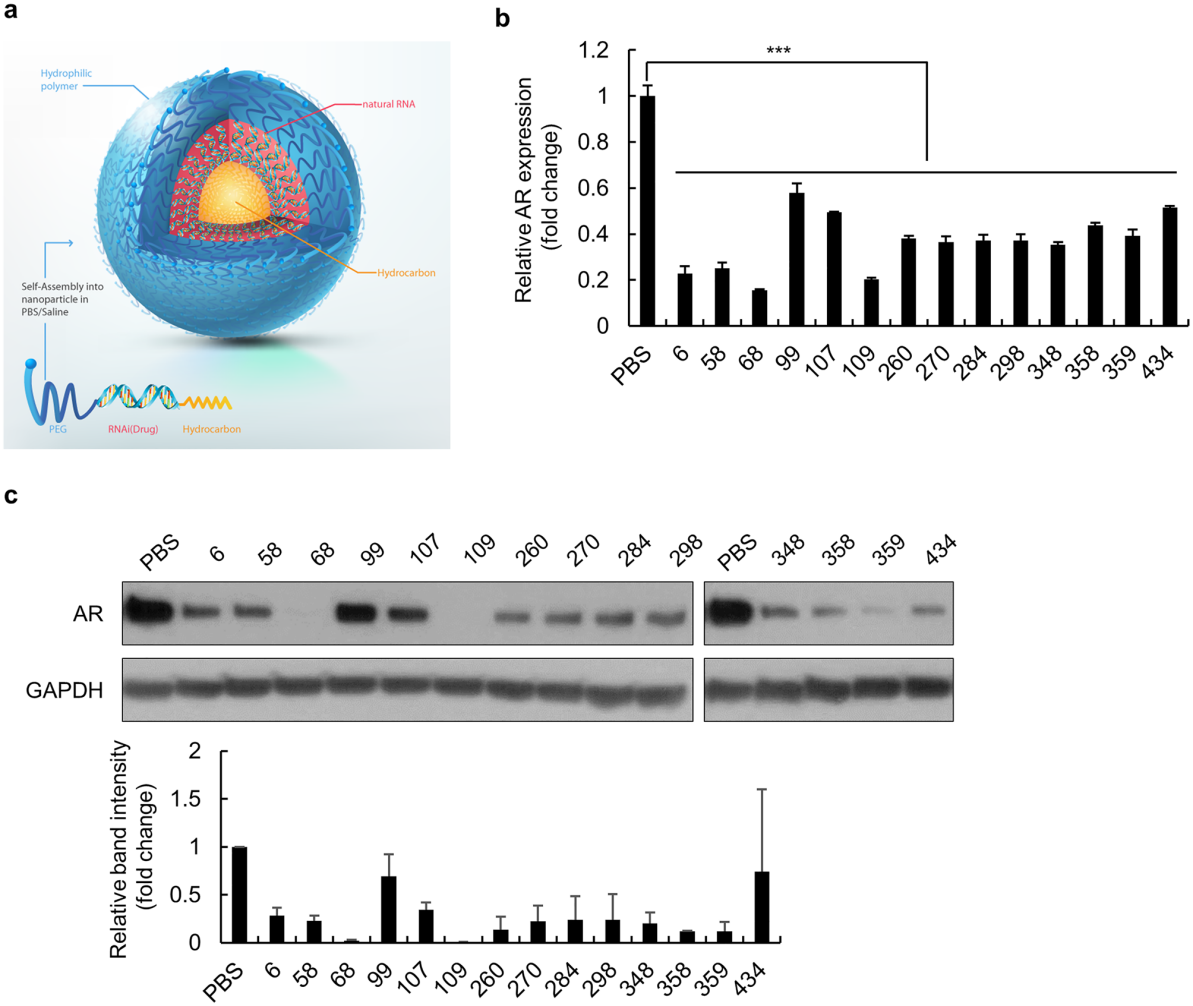
Screening of potent SAMiRNAs targeting human AR. (**a**) Schematic of SAMiRNA nanoparticles. (**b**, **c**) LNCaP cells were treated with PBS or 14 SAMiRNA candidates for 48 h. Total RNA extracts were subjected to quantitative polymerase chain reaction (qPCR) assays to evaluate AR knockdown efficacy, and AR expression was normalized to the expression of the ribosomal protein lateral stalk subunit P0 (RPLP0) gene (**b**). Whole-cell lysates were subjected to immunoblot analysis to compare the protein levels of AR and GAPDH. The intensity of the AR and GAPDH bands was quantified by ImageJ software, and AR protein expression was normalized to glyceraldehyde-3-phosphate dehydrogenase (GAPDH) values (**c**, lower panel). Statistical significance was assessed by two-sided Student’s *t* test, ***p < 0.001.

SAMiRNA nanoparticles, as shown in Fig. 1a, consist of hydrophilic polymer polyethylene glycol (PEG) and hydrophobic hydrocarbon conjugates at each end of an unmodified DNA/RNA heteroduplex. For potent SAMiRNAs specifically targeting AR, SAMiRNAs containing 19 base pairs of DNA/RNA heteroduplexes were designed by the sliding window algorithm and selected for specificity for AR mRNA. A total of 547 SAMiRNA candidate-conjugated sense strands and antisense strands were synthesized, purified and annealed to nanoparticles. The silencing effect of AR in LNCaP cells was analyzed for each SAMiRNA candidate by reverse transcription-quantitative polymerase chain reaction (RT-qPCR). Fourteen SAMiRNA candidates were selected based on their knockdown efficiency (> 50% AR silencing efficacy) (Supplementary Fig. S1 and Fig. 1b). In addition, we verified the decreased level of AR protein in fourteen SAMiRNA candidate-treated LNCaP cells (Fig. 1c). AR68 and AR109 were found to be the most potent SAMiRNAs.

**Figure 2:**
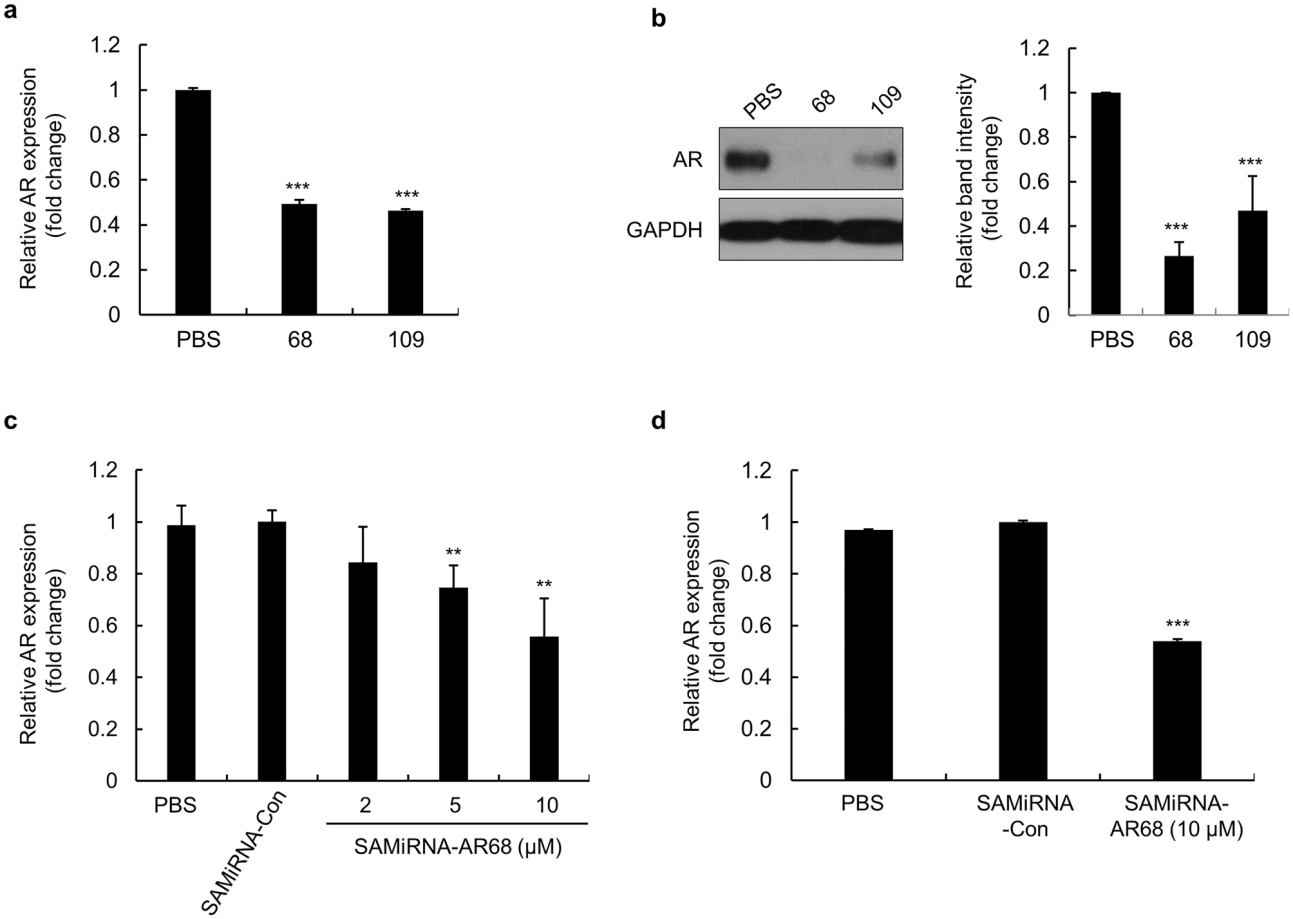
The efficacy of SAMiRNA-AR68 in reducing AR expression in HFDP cells. (**a**, **b**) Human follicle dermal papilla (HFPD) cells were treated with PBS, SAMiRNA-AR68, or SAMiRNA-AR109 for 48 h. Total RNA extracts were subjected to RT-qPCR assays, and AR expression was normalized to that of the RPLP0 gene (**a**). Whole-cell lysates were subjected to immunoblot analysis (**b**, left panel). The intensity of the AR and GAPDH bands was quantified by ImageJ software, and AR protein expression was normalized to GAPDH values (**b**, right panel). (**c**) HFDP cells were treated with PBS, SAMiRNA control, or the indicated doses of SAMiRNA-AR68 for 48 h. Total RNA extracts were subjected to qPCR assays, and AR expression was normalized to that of the RPLP0 gene. (**d**) HFDP cells were treated with PBS, SAMiRNA control, or 10 μM SAMiRNA-AR68 for 48 h. Whole-cell lysates were subjected to enzyme-linked immunosorbent assays (ELISAs) to measure AR protein. Statistical significance was assessed by two-sided Student’s *t* test, **p < 0.01; ***p < 0.001.

To confirm efficacy in human hair cells, we treated human follicle dermal papilla (HFDP) cells with AR68 and AR109, which significantly decreased AR mRNA and protein levels (Fig. 2a, b). As shown in Fig. 2b, AR68 reduced AR protein levels more effectively than AR109 in HFDP cells, confirming that AR68 is the more potent siRNA sequence for AR silencing. Treatment of HFPD cells with AR68 reduced AR mRNA expression in a dose-dependent manner (Fig. 2c), and the AR protein inhibitory effect was confirmed by enzyme-linked immunosorbent assays (ELISAs) (Fig. 2d).

### SAMiRNA-AR68 decreased the AR mRNA and protein levels in human hair follicles

**Figure 3:**
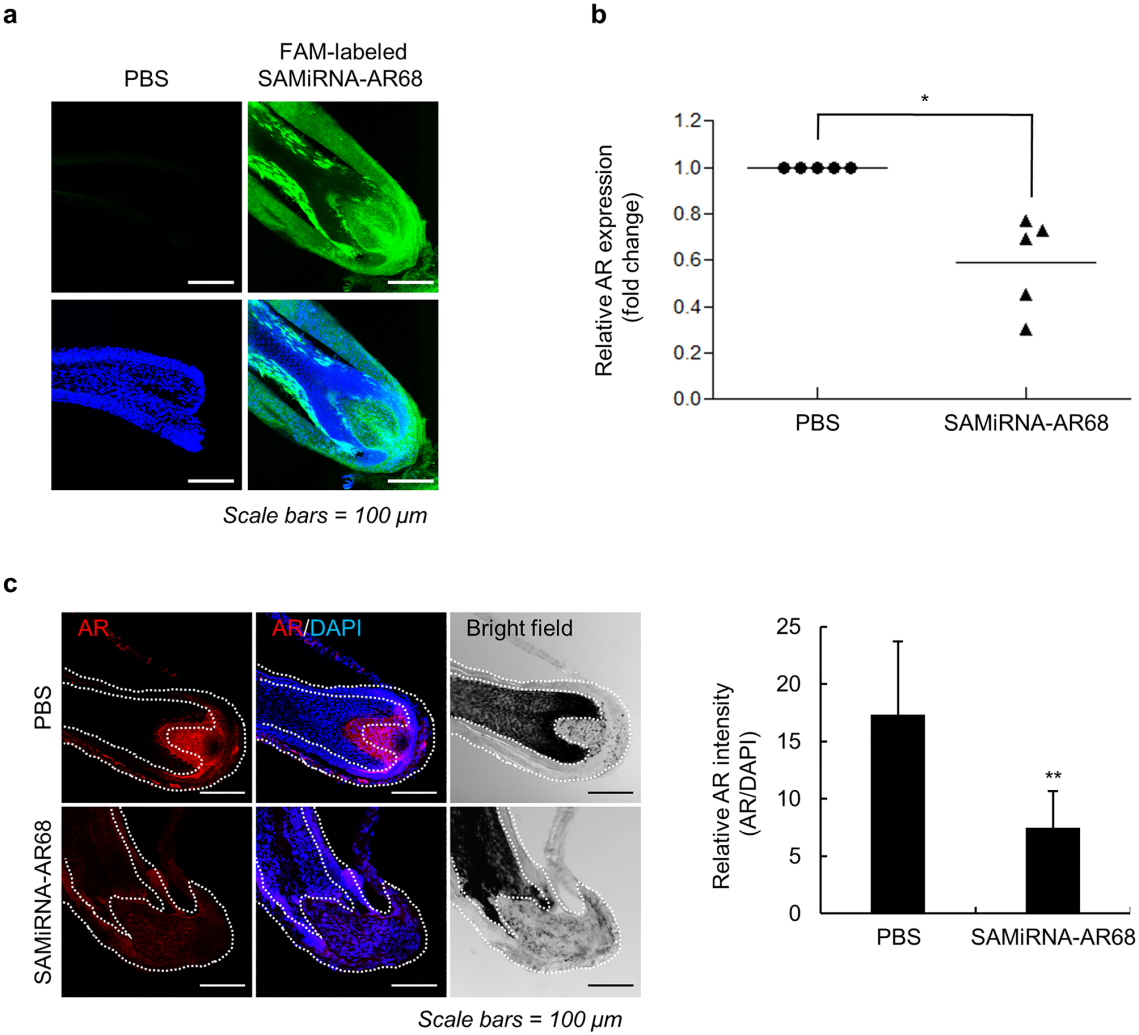
AR silencing efficacy of SAMiRNA-AR68 in human hair follicles. (**a**) Plucked human hair follicles were treated with PBS or 10 μM FAM-labeled SAMiRNA-AR68 in culture medium for 24 h. Hair follicles were subjected to immunofluorescence (IF) analysis and counterstained with 4′,6-diamidino-2-phenylindole (DAPI). Scale bars = 100 μm. (**b**) Plucked human hair follicles were treated with PBS or 10 μM SAMiRNA-AR68 for 48 h. Total RNA extracts from hair bulbs were subjected to qPCR assays, and AR expression was normalized to that of the RPLP0 gene. (**c**) Plucked human hair follicles were treated with PBS or 10 μM SAMiRNA-AR68 for 48 h. IF analysis of the plucked vertex hair follicle section incubated with an anti-AR antibody and counterstained with DAPI. Scale bars = 100 μm. Levels of AR protein were measured by the mean fluorescence intensity using ZEN software. Data are shown as the mean value ± SD (*n* = 8 hairs/group) normalized for DAPI intensity. Statistical significance was assessed by two-sided Student’s *t* test, *p < 0.05 and **p < 0.01.

To evaluate SAMiRNA nanoparticle delivery to human hair follicles, we applied fluorescein (FAM)-labeled AR68 to plucked hair and visualized the samples by confocal microscopy. Plucked hair often lacks the dermal papilla compared with microdissected hair follicles. We plucked multiple hairs and selected hairs with a hair bulb. FAM-labeled AR68 was added to plucked hairs with dermal papilla. As expected, FAM-labeled AR68 was efficiently delivered to the outer root sheath (ORS) as well as the dermal papilla of the hair bulb (Fig. 3a). We confirmed AR silencing in hair follicles by RT-qPCR analysis (Fig. 3b). Immunofluorescence analysis of the AR68-treated plucked hair showed a substantial reduction in AR protein expression (Fig. 3c). The AR protein is highly expressed in the dermal papilla, and transactivation of AR by binding to DHT in dermal papilla cells induces apoptosis that promotes AGA progression^2,25^. Therefore, we confirmed that AR68 nanoparticles at 10 µM could be delivered and silenced AR in dermal papilla cells of hair follicles. We investigated the efficacy of AR68 at higher concentrations for a clinical study of AGA treatment.

### The safety of SAMiRNA-AR68 nanoparticles

**Figure 4:**
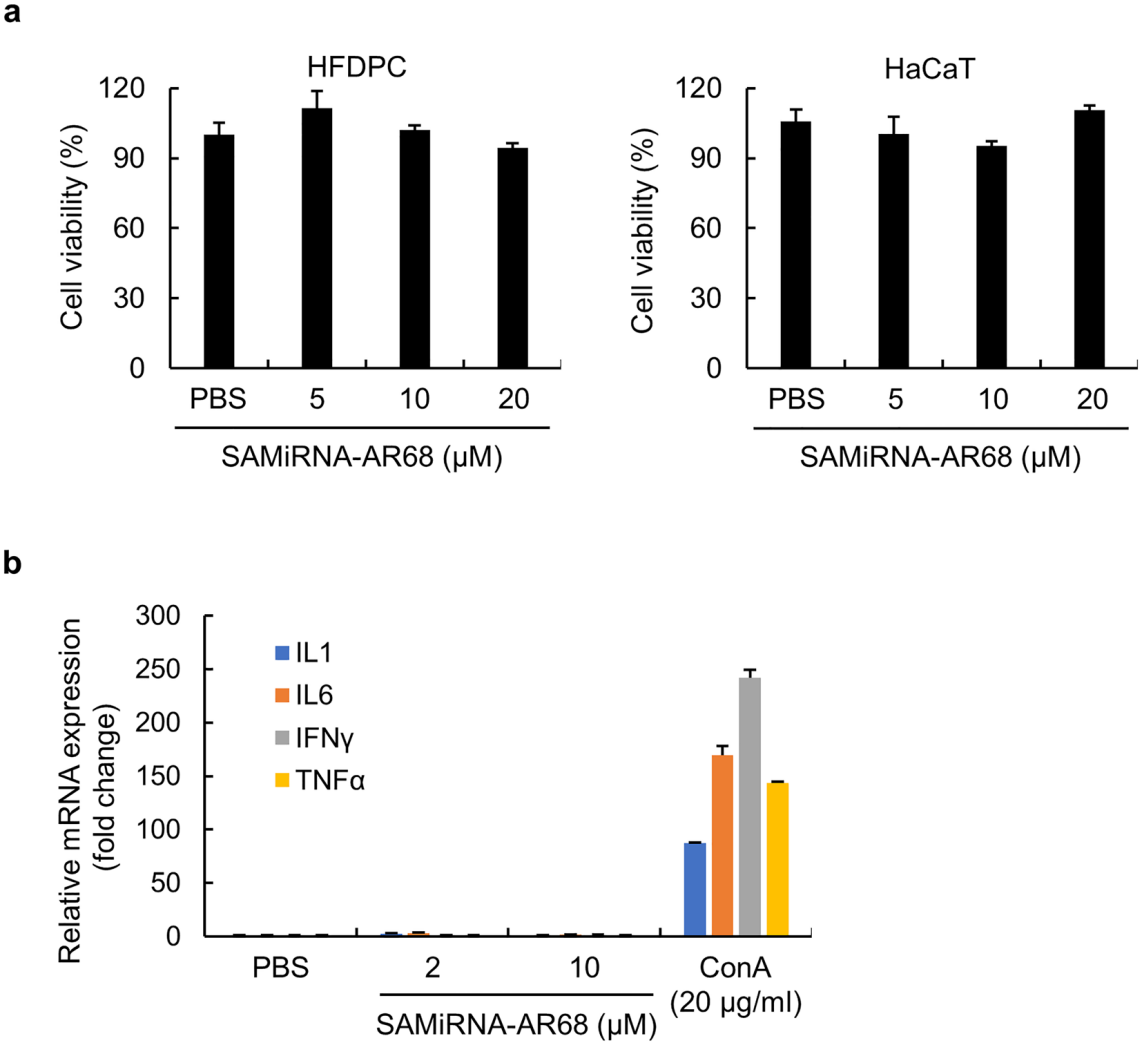
The safety of SAMiRNA-AR68. (**a**) HFDP and HaCaT cells were treated with PBS or the indicated dose of SAMiRNA-AR68 for 72 h. WST-1 assays were performed to measure cell viability. (**b**) Human PBMCs were treated with 20 μg/ml concanavalin A (ConA) as a positive control, PBS, or the indicated doses of SAMiRNA-AR68 for 6 h. Total RNA extracts were subjected to qPCR assays to evaluate the expression of proinflammatory cytokines, including IL-1β, IL-6, INF-γ and TNF-α, normalized to that of the ribosomal protein L13A (RPL13A) gene.

One of the limitations in developing RNAi therapeutics for clinical applications is cytotoxicity and systemic toxicity, including innate immune stimulation^34^. To evaluate the cytotoxicity of AR68, we performed a cell viability assay in HFDP and keratinocyte HaCaT cells at effective concentrations (10 μM). Neither HFDP nor HaCaT cells showed cytotoxicity up to 20 μM AR68 (Fig. 4a). We also evaluated whether AR68 induces nonspecific innate immune stimulation in human PBMCs. AR68 was treated at concentrations up to 10 μM, and proinflammatory cytokines related to TLR3 signaling were analyzed after 6 h. AR68 did not induce proinflammatory cytokines, including interleukin (IL)-1β, IL-6, interferon-gamma (INF-γ), and tumor necrosis factor-alpha (TNF-α), in PBMCs compared to nonstimulated negative controls; as a positive control, concanavalin A significantly induced proinflammatory cytokines (Fig. 4b). Taken together, these results demonstrate that AR68 does not induce cytotoxicity or innate immune stimulation at 10 µM, and these results guided determination of the effective dose of AR68 to be used in clinical applications.

### The stability of SAMiRNA-AR68 nanoparticles

We investigated the storage stability of SAMiRNA solution at various temperatures. The nanoparticle size of SAMiRNA was monitored by qNano Gold to evaluate the long-term stability of AR68 nanoparticles. The average size of the AR68 nanoparticles was approximately 99.2 ± 5.1 nm (22 °C, 55% ± 5 humidity) and 105.0 ± 2.5 nm (40 °C, 75% ± 5 humidity). The AR68 formulation remained stable for 6 months (Supplementary Fig. S2a). We further investigated AR knockdown by RT-qPCR using AR68 samples stored for 6 months under the indicated conditions. As expected, we observed a similar inhibitory effect of AR mRNA in HFDPCs (Supplementary Fig. S2b).

### Clinical study I: low-dose SAMiRNA-AR68 (0.5 mg/ml) treatment three times per week

#### General characteristics of the subjects

A total of 48 male and female subjects diagnosed with moderate androgenetic alopecia were recruited and randomly assigned to an AR68 low-dose treatment group (test group) (n = 24) or placebo group (n = 24). In the AR68 low-dose treatment group, 2 of 24 subjects dropped out (withdrawal of consent); thus, 22 subjects completed the study. The mean age was 42.4 ± 8.10 years, and the male to female ratio was 8:14. In the placebo group, one of 24 subjects dropped out (withdrawal of consent), with 23 completing the study. The mean age of the placebo group was 42.4 ± 6.69 years, and the male to female ratio was 6:17. There was no statistically significant difference between the two groups based on group homogeneity analysis performed to confirm the validity of randomization (Supplementary Fig. S3 and Table 1).

**Table 1:** Demographic characteristics of the subjects in clinical study I.

Characteristics		AR68 0.5 mg/ml (n = 22)	Placebo (n = 23)	*p* value
		n (%)	n (%)	
Sex	Male	8 (36.36)	6 (26.09)	0.530
	Female	14 (63.64)	17 (73.91)	

Age	Mean ± SD	42.4 ± 8.10	42.4 ± 6.69	0.546
	Median	44.00	43.00	
	Min, Max	22.00, 53.00	26.00, 53.00	
	20 <	0 (0.00)	0 (0.00)	
	20–29	2 (9.09)	1 (4.35)	
	30–39	3 (13.64)	6 (26.09)	
	40–49	14 (63.63)	13 (56.52)	
	≥ 50	3 (13.64)	3 (13.04)	

#### Analysis of hair density and total hair counts

**Figure 5:**
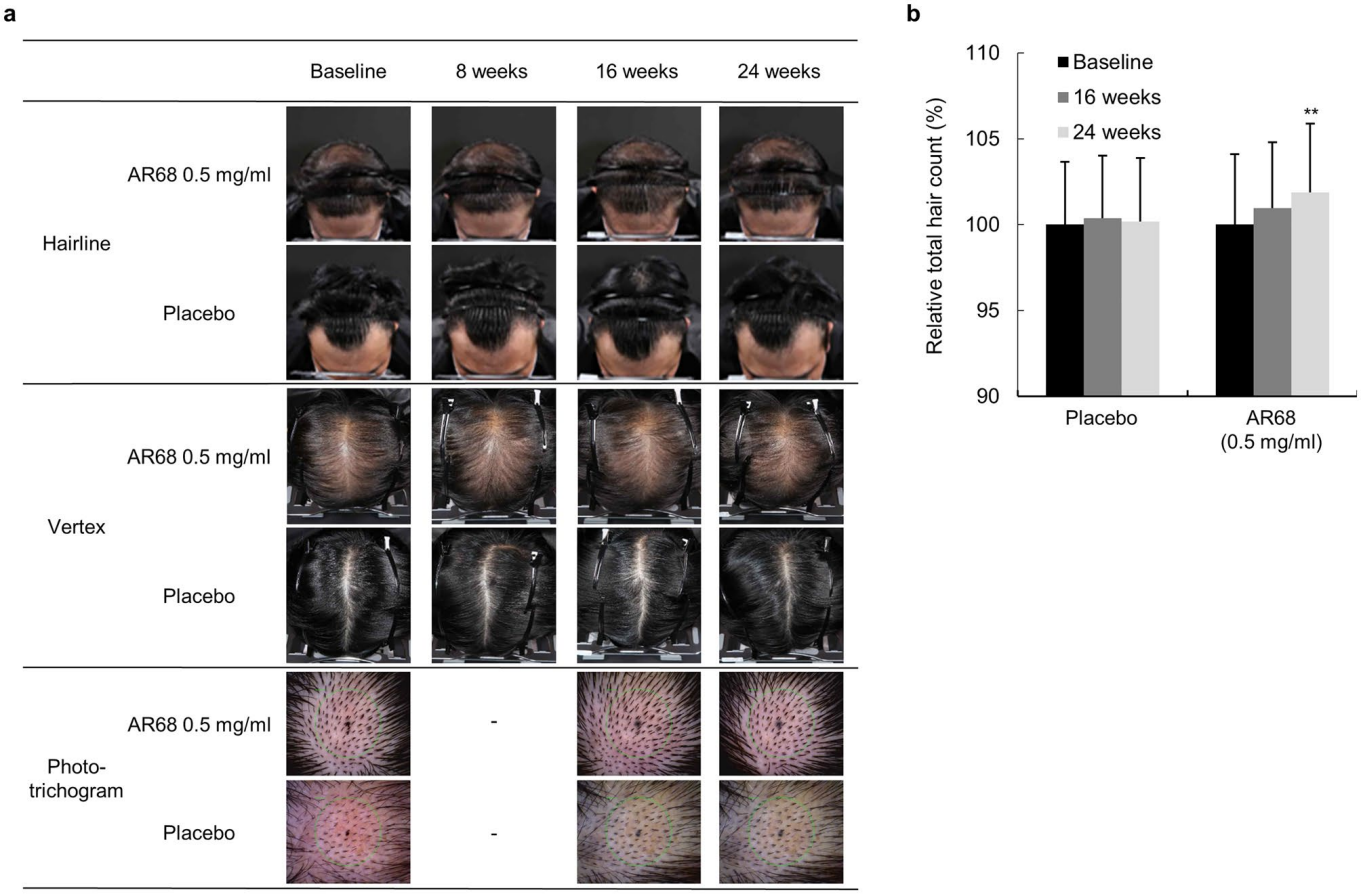
Images of the change in hair loss following 24 consecutive weeks of application of SAMiRNA-AR68 at 0.5 mg/ml and placebo products. (**a**) Representative photographs of the forehead hairline and vertex of the two groups at baseline and 8, 16 and 24 weeks after 0.5 mg/ml AR68 and placebo treatment three times per week. The phototrichogram analysis was performed at baseline and at 16 and 24 weeks. (**b**) Representative graph of the percentage of total hair count increments at 16 and 24 weeks compared to baseline.

Photographic assessment revealed increased hair density in the AR68 low-dose treatment group compared with that at baseline (Fig. 5a, Table 2a). As shown by phototrichogram analysis, the total hair count increased at 24 weeks after treatment with AR68 compared to that at baseline (from 133.14 to 135.41 hairs/cm^2^; *p* < 0.01) (Table 2b). The mean change and rate of total hair count increased by 2.273 ± 3.089 and 1.870% at 24 weeks in the AR68 0.5 mg/ml treatment group, respectively, compared to those at baseline (Fig. 5b, Table 3).

**Table 2:** Descriptive statistical analysis of hair density (a) and total hair counts (b) between time points in clinical study I.

	Baseline	8 weeks	16 weeks	24 weeks
**(a)**				
**AR68 0.5 mg/ml (n = 22)**				
Mean^a^	0.000	0.000	0.045	0.045
SD	0.000	0.000	0.213	0.375
*p* value	–	0.317	0.083	0.317
**Placebo (n = 23)**				
Mean	0.000	0.000	0.043	0.087
SD	0.000	0.000	0.209	0.417
*p* value	–	1.000	1.000	0.317
**(b)**				
**AR68 0.5 mg/ml (n = 22)**				
Mean (n/cm^2^)^b^	133.14	–	134.00	135.41
SD	25.64	–	24.21	25.48
*p* value^c^	–	–	0.078	0.002**
**Placebo (n = 23)**				
Mean (n/cm^2^)	129.26	–	129.70	129.57
SD	22.64	–	22.66	22.96
*p* value	–	–	0.441	0.588

^a^Increment of mean value represents improvement of hair condition on hairline and vertex.

^b^Increment of mean value represents improvement of hair counts.

^c^Significantly different at **p < 0.01 compared with baseline.

**Table 3:** Statistical analysis of Δ total hair counts (weeks-Baseline) between SAMiRNA-AR68 0.5 mg/ml treated and placebo groups.

Δ Total hair counts (n/cm^2^)	AR68 0.5 mg/ml (n = 22)	Placebo (n = 23)	*p* value^a^
	Mean ± SD	Mean ± SD	
16 weeks	0.864 ± 2.189	0.435 ± 2.660	0.477
24 weeks	2.273 ± 3.089	0.304 ± 2.653	0.043*

^a^Significantly different at *p < 0.05 compared with AR 0.5 mg/ml treated group and placebo group at same time point.

#### Subject self-assessment questionnaires

There was no significant difference between the AR68 0.5 mg/ml treatment group and the placebo group.

#### Safety assessments

In the safety assessment, no adverse events in any of the 45 subjects were observed during the clinical study period.

### Clinical study II: high-dose SAMiRNA-AR68 (5 mg/ml) treatment once a week

#### General characteristics of the subjects

To increase efficacy and user convenience, we designed and conducted a clinical study with high-dose (5 mg/ml) treatment once per week. A total of 60 male and female subjects diagnosed with moderate androgenetic alopecia participated and were randomly assigned to an AR68 5 mg/ml treatment group (n = 30) or a placebo group (n = 30). During the study period, six subjects withdrew (withdrawal of consent or loss to follow-up), ten subjects withdrew at the investigator's discretion (hair perm, dyeing, etc.), and one subject dropped out due to an adverse reaction; thus, 43 subjects completed the clinical study. In the AR68 treatment group, 8 of 30 subjects dropped out, and 22 completed the study. The average age was 44.77 ± 10.88 years, and the male to female ratio was 9:13. In the placebo group, 9 of 30 subjects dropped out, and 21 completed the study; the mean age was 46.48 ± 7.93 years, and the male to female ratio was 10:11. There was no statistically significant difference between the two groups, validating the randomization (Supplementary Fig. S4 and Table 4).

**Table 4:** Demographic characteristics of the subjects in clinical study II.

Characteristics		AR68 5 mg/ml (n = 22)	Placebo (n = 21)
		n (%)	n (%)
Sex	Male	9 (40.90)	10 (47.62)
	Female	13 (50.10)	11 (52.38)

Age	Mean ± SD	44.77 ± 10.88	46.48 ± 7.93
	Median	49.50	48.00
	Min, Max	22.00, 54.00	25.00, 54.00
	20 <	0 (0.00)	0 (0.00)
	20–29	4 (18.18)	2 (9.52)
	30–39	1 (4.55)	–
	40–49	6 (27.27)	9 (42.86)
	≥ 50	11 (50.00)	10 (47.62)

#### Analysis of total hair counts and photographic assessments

**Figure 6:**
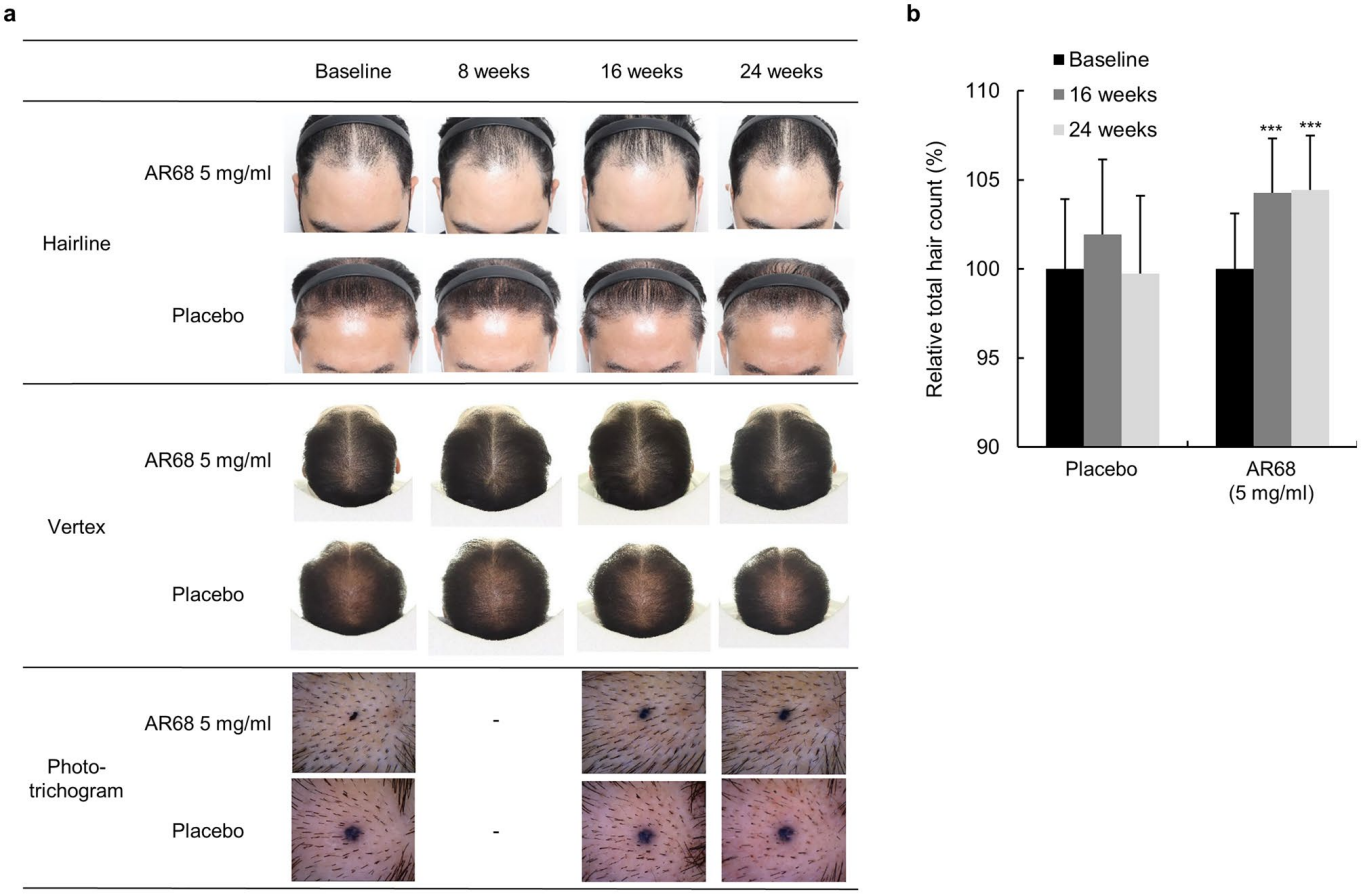
Images of the change in hair loss following 24 consecutive weeks of application of SAMiRNA-AR68 at 5 mg/ml and placebo products. (**a**) Representative photographs of the forehead hairline and vertex of the two groups at baseline and 8, 16 and 24 weeks after 5 mg/ml AR68 and placebo treatment once a week. The phototrichogram analysis was performed at baseline and at 16 and 24 weeks. (**b**) Representative graph of the percentage of total hair count increments at 16 and 24 weeks compared to baseline.

Hair density by photo assessment was significantly increased at eight weeks (0.091 ± 0.294), 16 weeks (0.159 ± 0.29; *p* < 0.05), and 24 weeks in the AR68 5 mg/ml treatment group compared to that at baseline (before the assessment). There was a significant improvement at week 24 (0.250 ± 0.551; *p* < 0.05) (Fig. 6a, Table 5a). Phototrichogram analysis showed significantly higher total hair counts for the AR68 treatment group at 16 weeks (from 182.182 to 189.727 hairs/cm^2^; *p* < 0.001) and 24 weeks (from 182.182 to 189.909 hairs/cm^2^; *p* < 0.001) (Table 4b) than that at baseline. The mean change and rate of total hair count increased by 7.545 ± 7.896 and 4.264% (*p* < 0.001) at 16 weeks and 7.727 ± 8.659 and 4.421% (*p* < 0.001) at 24 weeks in the AR68 5 mg/ml treatment group, respectively, compared to those at baseline (Fig. 6b, Table 6).

**Table 5:** Descriptive statistical analysis of hair density (a) and total hair counts (b) between time points in clinical study II.

	Baseline	8 weeks	16 weeks	24 weeks
**(a)**				
**AR68 5 mg/ml (n = 22)**				
Mean^a^	0.000	0.091	0.159	0.250
SD	0.000	0.294	0.358	0.551
**Placebo (n = 21)**				
Mean	0.000	− 0.095	− 0.071	− 0.071
SD	0.000	0.340	0.396	0.396
*p* value^c^	–	0.098	0.039*	0.035*
**(b)**				
**AR68 5 mg/ml (n = 22)**				
Mean(n/cm^2^)^b^	182.182	–	189.727	189.909
SD	26.626	–	27.216	27.154
*p* value^d^	–	–	< 0.001***	< 0.001***
**Placebo (n = 21)**				
Mean(n/cm^2^)	181.714	–	185.429	181.524
SD	32.661	–	35.778	36.371
*p* value	–	–	–	–

^a^Increment of mean value represents improvement of hair condition on hairline and vertex.

^b^Increment of mean value represents improvement of hair counts.

^c^Significantly different at *p < 0.05 compared with AR68 5 mg/ml treated group and placebo group at same time point.

^d^Significantly different at ***p < 0.001 compared with baseline.

**Table 6:** Statistical analysis of Δ total hair counts (weeks-Baseline) between SAMiRNA-AR68 5 mg/ml treated and placebo groups.

Δ Total hair counts (n/cm^2^)	AR68 5 mg/ml (n = 22)	Placebo (n = 21)	*p* value^a^
	Mean ± SD	Mean ± SD	
16 weeks	7.545 ± 7.896	3.714 ± 10.335	0.279
24 weeks	7.727 ± 8.659	− 0.190 ± 12.875	0.026*

^a^Significantly different at *p < 0.05 compared with AR68 5 mg/ml treated group and placebo group at same time point.

#### Subject self-assessment questionnaires

Regarding the efficacy of product use, the subjects in the AR68 5 mg/ml treatment group gave positive feedback in the subject self-assessment questionnaires. The participants responded that they were “satisfied with the sample used” at 8 (72.73%), 16 (81.82%), and 24 (59.09%) weeks. At weeks 8 (36.36%), 16 (63.64%) and 24 (50.00%), “the feeling of fuller hair in the vertex area” was reported, and hair loss decreased at weeks 8 (45.45%), 16 (54.55%) and 24 (72.73%).

#### Safety assessments

In the safety assessment, one subject in the AR68 5 mg/ml treatment group developed erythema, edema, and itching at the test site, but the symptoms were relieved after diagnosis and treatment by a dermatologist. The dermatologist's diagnosis determined that the relationship between the symptoms and the test substance was slight.

## Discussion

This study demonstrates that inhibiting AR expression using SAMiRNA nanoparticles targeting human AR mRNA is an effective method for AGA treatment. AR68 treatment significantly reduced the AR mRNA and protein levels in HFDPCs and human hair follicles. In two clinical studies, quantitative phototrichogram analysis confirmed that the total hair count increased in the AR68-treated test group but decreased in the placebo group.

Although siRNA therapeutics still have some limitations, including nonspecific innate immune stimulation^32–34^, AR68 showed efficacy and safety in HFDPCs and human PBMCs. This treatment did not induce inflammatory cytokines at the effective dose. Our group recently reported the preclinical toxicity results of another SAMiRNA for fibrosis treatment. After intravenous administration, the no observed adverse effect level (NOAEL) in mice and non-human primates (NHPs) were determined to be 300 mpk and 100 mpk or higher, respectively^43,44^. SAMiRNA is thus a very safe siRNA modality.

For the development of AGA treatment using AR68, AR68 (Cosmerna-68) is listed as a cosmetic ingredient in cosmetic ingredients (ICID) and the Cosmetic Ingredient Dictionary, which are reviewed by the PCPC (Personal Care Products Council) and KCA (Korea Cosmetic Association), respectively. The stability of AR68 nanoparticles in a hair tonic formulation containing cosmetic ingredients was studied by measuring the size of AR68 nanoparticles that remained stable for more than six months.

Before the clinical study, a primary dermal irritation study was performed on 35 healthy volunteers (13 male and 22 female). Twenty microliters of the formulation containing AR68 at 0.5 mg/ml or 5 mg/ml was administered to a patch applied to the back and removed at 30 min and 24 h, and no skin dermal reaction was observed (data not shown).

In low-dose (0.5 mg/ml) clinical study I, the total hair count of the AR68 treatment group increased by 1.870% (from 133.14 to 135.41 hairs/cm^2^) at 24 weeks after application three times per week. To increase efficacy and user convenience, we performed high-dose (5 mg/ml) treatment once per week for 24 weeks in clinical study II. The high-dose (5 mg/ml) AR68 treatment group’s total hair counts increased significantly to 4.421% (from 182.182 to 189.909 hairs/cm^2^) after 24 weeks, whereas the placebo group’s total hair counts decreased slightly at 24 weeks (from 181.714 to 181.524 hairs/cm^2^). The AR68 high-dose treatment group showed approximately 240% efficacy compared with the low-dose group (7.727 and 2.273 total hair counts, respectively) at 24 weeks, despite one application per week. Although low-dose AR68 treatment was applied three times per week, it did not result in remarkable improvement. In the photographic assessment score, there was no statistically significant difference between the placebo group and the 0.5 mg/ml AR68 treatment group compared to the baseline, but in the 5 mg/ml AR68 treatment group, a significant improvement was confirmed at 16 weeks (0.159 ± 0.358) and 24 weeks (0.250 ± 0.551). In the high-dose AR68 group, the total hair count increased significantly at 24 weeks compared to that at the baseline (4.421%; *p* < 0.001). Additionally, many of the high-dose AR68 group subjects expressed satisfaction in the self-assessment questionnaire. As the activity of siRNA is maintained for a long time due to an RNA-induced silencing complex (RISC) in cells, efficient delivery is more important than frequent applications. We confirmed through two independent clinical studies that a high-dose single application per week is much more potent than low-dose frequent applications. Weekly application is a convenient benefit of SAMiRNA for AGA treatment. In the safety assessment, one subject in the high-dose AR68-treated group had an adverse reaction, but it was confirmed that the symptoms were not caused by the test substance. The other subjects had no adverse effects during 24 weeks of treatment, and the safety of AR68 in scalp treatment was confirmed.

This study demonstrates the efficacy and safety of SAMiRNA-AR68 through an in vitro analysis of SAMiRNA-AR68 and two clinical studies. In comparison to the ~9.3 hairs/cm^2^ average hair growth seen with daily oral administration of 1 mg finasteride over a 24-week period^45–47^, high-dose AR68 treatment per week over 16-week period led to average hair growth of 7.5 hairs/cm^2^, indicating comparable efficacy as finasteride. AR68 has no adverse effects and is more convenient as a weekly treatment. This study has limitations in a number of subjects and a spectrum of races. We plan to perform a clinical study with Caucasian subjects with a higher AGA incidence. In conclusion, SAMiRNA-AR68 is a promising new treatment for AGA.

## Methods

### Cell culture and regents

Human prostate cancer LNCaP and human keratinocyte HaCaT cells were purchased from the American Type Culture Collection (ATCC), and human follicle dermal papilla cells (HFDPCs) were purchased from PromoCell (C-12071). Human peripheral blood mononuclear cells (PBMCs) were obtained from Cellular Technology Limited (CTL-UP1). LNCaP cells were cultured in RPMI medium (HyClone) supplemented with 1% penicillin–streptomycin (HyClone) and 10% fetal bovine serum (FBS, HyClone). HaCaT cells were cultured in DMEM (HyClone) supplemented with 1% penicillin–streptomycin and 10% FBS. HFDPCs were maintained in follicle dermal papilla cell growth medium (PromoCell). Individual human hairs were pulled out with forceps in the occipital area of the scalp, and plucked hairs with visible bulbs were selected by microscopy. The plucked hair follicles were cultured in DMEM/F12 (Gibco) supplemented with 1% penicillin–streptomycin, 10% FBS, 10 µg/mL insulin-transferrin-selenium-X supplement (Gibco), 2.5 µg/mL amphotericin B (Gibco), 1% GlutaMAX (Gibco), 20 ng/mL fibroblast growth factor (FGF, PeproTech), 20 ng/mL epidermal growth factor (EGF, Sigma), and 10 ng/mL hydrocortisol (Tokyo Chemical Industry). Plucked human hair follicles were treated with 10 μM SAMiRNA-AR68 for 48 h followed by qPCR and immunofluorescence analysis. The synthesis and quality control of the SAMiRNA nanoparticles were previously described^35,37^.

### Measurement of SAMiRNA nanoparticle size

For determination of the long-term stability of SAMiRNA, the nanoparticle size was monitored by qNano Gold (Izon Science) according to standard operating procedures. Briefly, 35 μl of SAMiRNA-AR68 was analyzed with qNano Gold equipment using an NP80 Nanopore (Izon Science) and applying a 47 mm stretch, a current of 140 nA, and 10 mBar parametric conditions. The calibration particles (CPC100, Izon Science) were assayed before the experimental samples under identical conditions. Particle counts (≥ 50 events each) were finally determined using the qNano software provided by Izon Science (Izon Control Suite Version 3.3).

### Reverse transcription and quantitative polymerase chain reaction (RT-qPCR)

RT-qPCR was performed according to the MIQE guidelines^48^. Total RNA from cells was extracted using an *AccuPrep*® Universal RNA Extraction Kit (K-3140, Bioneer) according to the manufacturer’s instructions. For total RNA extraction from hair follicles, SAMiRNA-AR68-treated plucked hair follicles were washed with PBS, resuspended in TRIzol® reagent (Invitrogen), homogenized with a Biomasher II® Disposable Micro Tissue Homogenizer (Polyscience), and purified with an AccuPrep® Universal RNA Extraction Kit (K-3140, Bioneer) according to the manufacturer’s instructions. Total RNA (1 μg) was reverse transcribed using *Accupower*® *RocketScript*™ Cycle RT PreMix (dT20) in a 20 μl reaction (K-2201, Bioneer) according to the manufacturer’s instructions. For qPCR analysis, 10 μl of tenfold diluted cDNA was amplified using *AccuPower*® 2X *GreenStar*™ qPCR MasterMix (K-6253, Bioneer). The following primer sets were used: *AR*, forward 5′-TTGTACACGTGGTCAAGTGG-3′ and reverse 5′-TGGAGTTGACATTGGTGAAGG-3′; *RPLPO*, forward 5′-TGCCATTGCCCCATGTGAAG-3′ and reverse 5′-AGCTGCACATCACTCAGGATT-3′; *IL-1B*, forward 5′-CTGAGCTCGCCAGTGAAAT-3′ and reverse 5′-CTGTAGTGGTGGTCGGAGA-3′; *IL-6*, forward 5′-AGATGCAATAACCACCCCTG-3′ and reverse 5′-TGCGCAGAATGAGATGAGTT-3′; *TNF*, forward 5′-CTGTAGCCCATGTTGTAGCA-3′ and reverse 5′-GGTTATCTCTCAGCTCCACG-3′; *IFNG*, forward 5′-GAATGTCCAACGCAAAGCAA-3′ and reverse 5′-ACCTCGAAACAGCATCTGAC-3′; *RPL13A*, forward 5′-TGCCATTGCCCCATGTGAAG-3′ and reverse 5′-AGCTGCACATCACTCAGGATT-3′; *IL-1B*, forward 5′-GTGTTTGACGGCATCCCACC-3′ and reverse 5′-TAGGCTTCAGACGCACGACC-3′. PCR amplification was performed in a 50 μl reaction as follows: one cycle at 95 °C for 10 min, followed by 40 cycles at 95 °C for 5 s, 58 °C for 25 s, and 72 °C for 30 s, with one final extension step at 72 °C for 5 min. All experiments were performed in triplicate. The delta-delta Ct method was used to determine relative fold changes, and all data were normalized to the internal control gene.

### Immunoblot analysis

LNCaP and HFDP cells were collected and lysed using cell lysis buffer (Cell Signaling Technology) with protease inhibitor cocktail (Thermo Fisher Scientific). Proteins were separated by 10% sodium dodecyl sulfate–polyacrylamide gel electrophoresis (SDS-PAGE) and transferred to a PVDF membrane (Bio-Rad). After the membranes were blocked with 5% skim milk in Tris-buffered saline (TBS) for at least 1 h, they were incubated with the indicated antibodies overnight, washed with TBS three times, and then incubated with horseradish peroxidase-conjugated secondary antibodies for 2 h. Immunoblotting was performed with the following antibodies: anti-AR (ab133273, Abcam), anti-GAPDH (2118, Cell Signaling Technology), and horseradish peroxidase-conjugated anti-rabbit (7074, Cell Signaling Technology). Immunoblot band intensities of AR and GAPDH were quantified by ImageJ software, and the level of AR protein expression was normalized to the GAPDH values.

### Enzyme-linked immunosorbent assay (ELISA)

ELISA to detect the AR protein was performed using a human AR ELISA kit (LS-F4505, LSBio) according to the manufacturer’s manual. In brief, HFDP cells (5 × 10^4^ cells/well) were collected and lysed using cell lysis buffer (Cell Signaling Technology), and then, 100 μl of cell lysate was added to each well for 1 h at 37 °C. After incubation, a biotin-conjugated detection antibody (detection reagent A) was added that bound to the captured antigen. An avidin-horseradish peroxidase conjugate (detection reagent B) that binds to biotin was then added. The TMB substrate reacts with the HRP enzyme, resulting in color development. A sulfuric acid solution (stop solution) terminated the color development reaction, and the optical density (OD) of each well was measured at a wavelength of 450 nm. The OD of samples was computed with an OD standard curve generated using known standard AR concentrations (0.313 ~ 20 ng/ml) to determine its AR concentration. The incubation and washing steps were performed according to the supplier’s manual. Absorbance at 450 nm was monitored with a FLUOstar Omega microplate reader (BMG Labtech).

### Cell viability assay

For the cell viability assay, HaCaT and HFDP cells were seeded at a density of 4 × 10^3^ cells/well in a 96-well plate and then treated with the indicated conditions for 72 h. After incubation, 10 μl of WST (water soluble tetrazolium salt) reagent (EZ-Cytox, DoGen) was added to each well for 30 min at 37 °C. Absorbance at 450 nm was monitored with a FLUOstar Omega microplate reader (BMG Labtech).

### Immunofluorescence (IF)

Plucked hairs from healthy donors were obtained from the occipital area of the scalp. After incubation, hair follicles were washed three times with 0.05% Tween-20 (Sigma) in PBS for 5 min and permeabilized in 0.1% Triton X-100 (Sigma) in PBS for 1 min. Hair follicles were fixed in 4% formaldehyde (Sigma) in PBS for 20 min, placed on a cryotray and covered with OCT compound (Tissue-Tek). The mold was slowly placed in liquid nitrogen until the entire tissue block was completely frozen. The frozen tissue blocks were sectioned at 10 µm using a cryotome (Leica Biosystems). Hair follicle sections were incubated overnight at 4 °C with an anti-AR antibody (ab133273, Abcam). The sections were subsequently incubated with anti-rabbit Fluor 568 (A11011, Invitrogen) secondary antibody for 1 h at room temperature. After at least three washes, the sections were counterstained with DAPI (D9542, Sigma-Aldrich) and mounted in Immu-Mount solution (Thermo Fisher Scientific). All incubations were conducted in dark and humid chambers. The fluorescence signal was visualized using a confocal microscope (LSM880, Carl Zeiss) at excitation wavelengths of 568 nm (Alexa Fluor 568) and 405 nm (DAPI). At least three fields per section were analyzed. ZEN software (Zeiss) was used to analyze images of the desired area to measure the intensity on a pixel-by-pixel basis and calculate the mean intensity automatically.

### Statistical analysis

All data are presented as the mean ± standard deviation (SD), and the number of samples is indicated in each figure legend. The statistical significance of differences was assessed using the two-sided Student’s *t* test. The results shown are representative of at least three independent experiments. Significance was denoted as *p < 0.05, **p < 0.01, and ***p < 0.001.

### Clinical study I: low-dose SAMiRNA-AR68 (0.5 mg/ml) treatment three times per week

Clinical study I was a double-blind, randomized, placebo-controlled study in accordance with the Korea Ministry of Food and Drugs Safety (MFDS) Guidelines on the Efficacy Study of Cosmetics for Hair Loss Relief (July 2018). The study was approved by the Institutional Review Board of the local committee, Seowon Skin Research Center, and was conducted on 10/09/2020 (IRB No. 1040820–202,009-HR004–02) and all experimental protocols were carried out in accordance with relevant guidelines and regulations. All subjects provided written informed consent for publication of identifying information/images in an online open-access publication prior to study participation. Summary information of the study is listed and available at CRIS Registration No. KCT0005501: https://cris.nih.go.kr/cris/search/detailSearch.do/20301. Subjects were selected on the basis of inclusion and exclusion criteria and consisted of 48 Korean males and females ranging from 22 to 53 years of age. Male subjects were diagnosed with more than the M1, C1 and U1 range as the basic type and more than the V1 and F1 range as the specific type according to the basic and specific (BASP) classification^49^. Female subjects were diagnosed with more than the F1 and L1 ranges according to the BASP and Ludwig classification, respectively. The exclusion criteria were participation in a previous study within 6 months, prior surgical correction of scalp hair loss, use of topical minoxidil or finasteride within 6 months, and other skin diseases of the scalp, including severe seborrheic dermatitis, psoriasis, lichenoid eruption or other scalp infections. Alterations in hair styling and dyeing of the hair were not allowed during the study.

### Clinical study II: high-dose SAMiRNA-AR68 (5 mg/ml) treatment once a week

The second clinical study was conducted as a double-blind, randomized, placebo-controlled study in accordance with the Korea MFDS Guideline on the Efficacy Study of Cosmetics for Hair Loss Relief (July 2018). A summary of the study details is available through the following link: https://cris.nih.go.kr/cris/search/detailSearch.do/20367 (CRIS Registration No. KCT0005618). Clinical study II was approved by the Institutional Review Board of the local committee, Ellead Skin Research Center, and was conducted on 07/09/2020 (IRB No. 200804T001) and all experimental protocols were carried out in accordance with relevant guidelines and regulations. All subjects provided written informed consent for publication of identifying information/images in an online open-access publication prior to study participation. The subjects were selected on the basis of inclusion and exclusion criteria and consisted of 60 Korean males and females ranging from 22 to 54 years of age. The AR68 high-dose (5 mg/ml) formulation was the same as the composition used in clinical study I, and randomly blinded products were applied once per week after shampooing. The measurement and analysis of hair density and total hair counts were conducted using the same protocol as described for clinical study I. The total hair count was measured using a Folliscope® 5.0 phototrichogram system (LeadM). Statistical analysis was also performed using the same program, statistical software SPSS statistics version 26.0 (IBM), as in clinical study I.

### SAMiRNA-AR68 formulation and treatment

SAMiRNA-AR68 was formulated as a hair tonic aqueous solution with ethanol (15%, v/v), niacinamide (1% w/v), betaine (1% w/v), biotin (0.02% w/v) and buffer. The AR68 hair tonic was packaged in a piston-type bottle with a silicon adaptor for massage (Supplementary Fig. S5). The placebo was prepared in the same manner as the formula except for the addition of AR68 and packaged in the same bottle. In clinical study I, randomly selected products (AR68, 0.5 mg/ml) were used three times per week after shampooing, whereby the scalp was massaged for 5 min after application. In clinical study II, randomly selected products (AR68, 5 mg/ml) were used once a week after shampooing, whereby the scalp was massaged for 5 min after application. Product usage and compliance were monitored for each subject by reviewing subject diaries and weighing the returned test products at each scheduled visit (weeks 8, 16 and 24).

### Measurement of hair density and total hair count

All subjects placed their head into a hair photograph device (Canfield Scientific), and photographs were taken at fixed distance, angle and lighting with an EOS Rebel T6i digital camera (Canon). Global evaluation was performed by comparing clinical images at baseline with those at 8, 16 and 24 weeks after treatment with the test product. The investigator qualitatively assessed clinical images on a 7-point scale (− 3, marked decrease; − 2, intermediate decrease; − 1, slight decrease; 0, no change; + 1, slight increase; + 2, intermediate increase; and + 3, marked increase).

For evaluation of total hair counts, the hair was clipped approximately 2 mm after a red spot tattoo in the evaluation area (1 cm^2^) of the hair loss region (forehead hairline or vertex). The total hair counts were assessed using a Folliscope® 2.8 phototrichogram system (magnification 14 times, LeadM) at baseline and at 16 weeks and 24 weeks after treatment. The total hair count (number/cm^2^) was calculated as the number of hairs within an area^50^.

### Subject self-assessment questionnaires

Subject self-assessment questionnaires on efficacy and safety at 8 weeks, 16 weeks, and 24 weeks after using the product were collected.

### Safety assessments

Dermatologists observed and assessed the subjects for the occurrence of objective irritation, such as edema, itching, and burning. The details, including start and end dates of occurrence, degree of severity, treatment, and causal relationship with the test product, were recorded on the form. If any adverse reaction occurred, the dermatologist’s assessment of the adverse reaction was further conducted in accordance with the standard operating procedures for adverse reactions.

### Statistical analysis

Data are expressed as the mean and a changing rate between the baseline value and the value obtained at each time point. All statistical analyses were performed using the SPSS® package (IBM). For photographic assessment of hair density, statistical analysis for variables for comparison between time points or groups was conducted using the Wilcoxon signed-rank test and Mann–Whitney U test (*p* < 0.05). For analysis of total hair counts, the significance of a difference between time points was calculated using the paired t test, with *p* < 0.05 as the significance level.

## Supplementary Information

Supplementary Information.
