# Author Correction: Weekly treatment with SAMiRNA targeting the androgen receptor ameliorates androgenetic alopecia

**DOI:** 10.1038/s41598-022-10024-2

**Published:** 2022-04-05

**Authors:** Sung-Il Yun, Sang-Kyu Lee, Eun-Ah Goh, Oh Seung Kwon, Woorim Choi, Jangseon Kim, Mi Sun Lee, Soon Ja Choi, Seung Sik Lim, Tae Kee Moon, Sin Hae Kim, Keeyeol Kyong, Gaewon Nam, Han-Oh Park

**Affiliations:** 1 Bioneer Corporation, 8-11 Munpyeongseo-ro, Daedeok-gu, Daejeon, 34302 Republic of Korea; 2 siRNAgen Therapeutics, Daejeon, 34302 Republic of Korea; 3 Ellead Skin Research Center, Ellead, Seongnam, 13590 Republic of Korea; 4 Department of Bio-Cosmetics, Seowon University, 377-3 Musimseoro, Seowon-gu, Cheongju, 28674 Republic of Korea

Correction to: *Scientific Reports* 10.1038/s41598-022-05544-w, published online 31 January 2022

The original version of this Article contained errors.

In the Abstract,

“In the high-dose (5 mg/ml) clinical study, AR68 was given once per week for 24 weeks and showed 83% efficacy in increasing hair counts compared with finasteride.”

Now reads:

“In the 24-week long high-dose (5 mg/ml) clinical study, AR68 showed average additional hair growth of 1.3–1.9 hairs/cm^2^ per month, which is comparable to finasteride.”

In the Discussion section,

“In comparison with the clinical trial of daily oral administration of 1 mg finasteride for a 24-week period^45,46,47^, high-dose AR68 treatment per week caused 83% improvement in hair loss. Although the efficacy did not reach that of finasteride, AR68 has no adverse effects and is more convenient as a weekly treatment. This study has limitations in a number of subjects and a spectrum of races. For further studies, we will test lower frequencies treatment through clinical studies with more subjects to determine the best dose and frequency. In further studies, we will analyze androgen levels in FPHL to determine the relationship between the efficacy of AR68 and androgen levels. We also plan to perform a clinical study with Caucasian subjects with a higher AGA incidence.”

Now reads:

“In comparison to the ~9.3 hairs/cm^2^ average hair growth seen with daily oral administration of 1 mg finasteride over a 24-week period^45,46,47^, high-dose AR68 treatment per week over 16-week period led to average hair growth of 7.5 hairs/cm^2^, indicating comparable efficacy as finasteride. AR68 has no adverse effects and is more convenient as a weekly treatment. This study has limitations in a number of subjects and a spectrum of races. We plan to perform a clinical study with Caucasian subjects with a higher AGA incidence.”

The original Article has been corrected.

