# Supplementary material for: Weekly treatment with SAMiRNA targeting the androgen receptor ameliorates androgenetic alopecia
Source: Sci Rep. 2022 Jan 31;12:1607. doi: 10.1038/s41598-022-05544-w (PMC8803970; doi:10.1038/s41598-022-05544-w)

Weekly treatment with SAMiRNA targeting the androgen receptor ameliorates androgenetic alopecia

Sung-Il Yun[1,5], Sang-Kyu Lee[2,5], Eun-Ah Goh[2], Oh-Seung Kwon[2], Woorim Choi[2], Jangseon Kim[2], Mi Sun Lee[2], Soon Ja Choi[2], Seung Sik Lim[1], Tae Kee Moon[3], Sin Hae Kim[3], Keeyeol Kyong[4], Gaewon Nam[4*], and Han-Oh Park[1,2*]

[1]Bioneer Corporation, Daejeon 34302, Republic of Korea; [2]siRNAgen Therapeutics, Daejeon 34302, Republic of Korea; [3]Ellead Skin Research Center, Ellead, Seongnam 13590, Republic of Korea; [4]Department of Bio-cosmetics, Seowon University, Cheongju 28674, Republic of Korea; [5]These authors contributed equally: Sung-Il Yun and Sang-Kyu Lee.

Correspondence: Han-Oh Park, Bioneer Corporation, 8-11 Munpyeongse-ro, Daeduck-gu, Daejeon 34302, Republic of Korea, Tel: +82-42-930-8500, Fax: +82-42-930-8600, E-mail: hpark@bioneer.com

Gaewon Nam, Seowon University, 377-3 Musimseoro, Seowon-gu, Cheongju 28674, Republic of Korea, Tel: +82-43-299-8220, Fax: +82-43-299-8223, E-mail: skarod@seowon.ac.kr

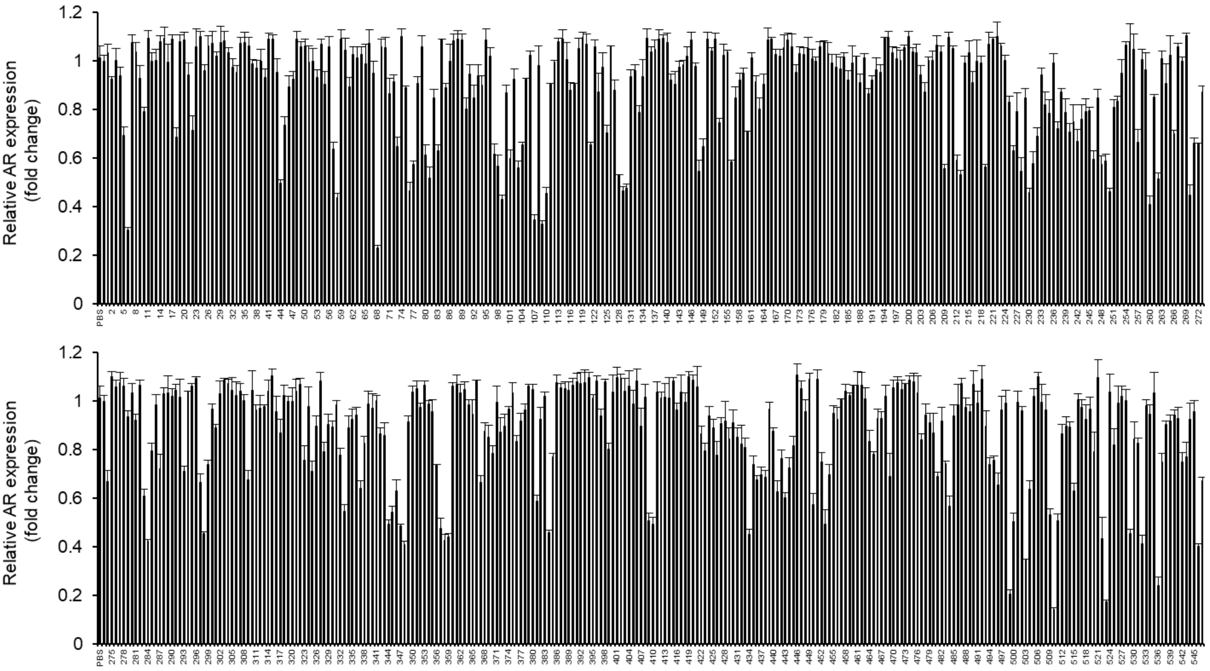

Supplementary Figure S1. Screening of SAMiRNA containing potent siRNAs targeting AR mRNA from 547 siRNA candidates. LNCap cells were treated with PBS or SAMiRNA containing indicated 547 siRNAs each for 48 hr. The total RNA extracts were subjected to qPCR assays to evaluate the AR knockdown efficacy, and AR expression was normalized to that of the RPLP0 gene.

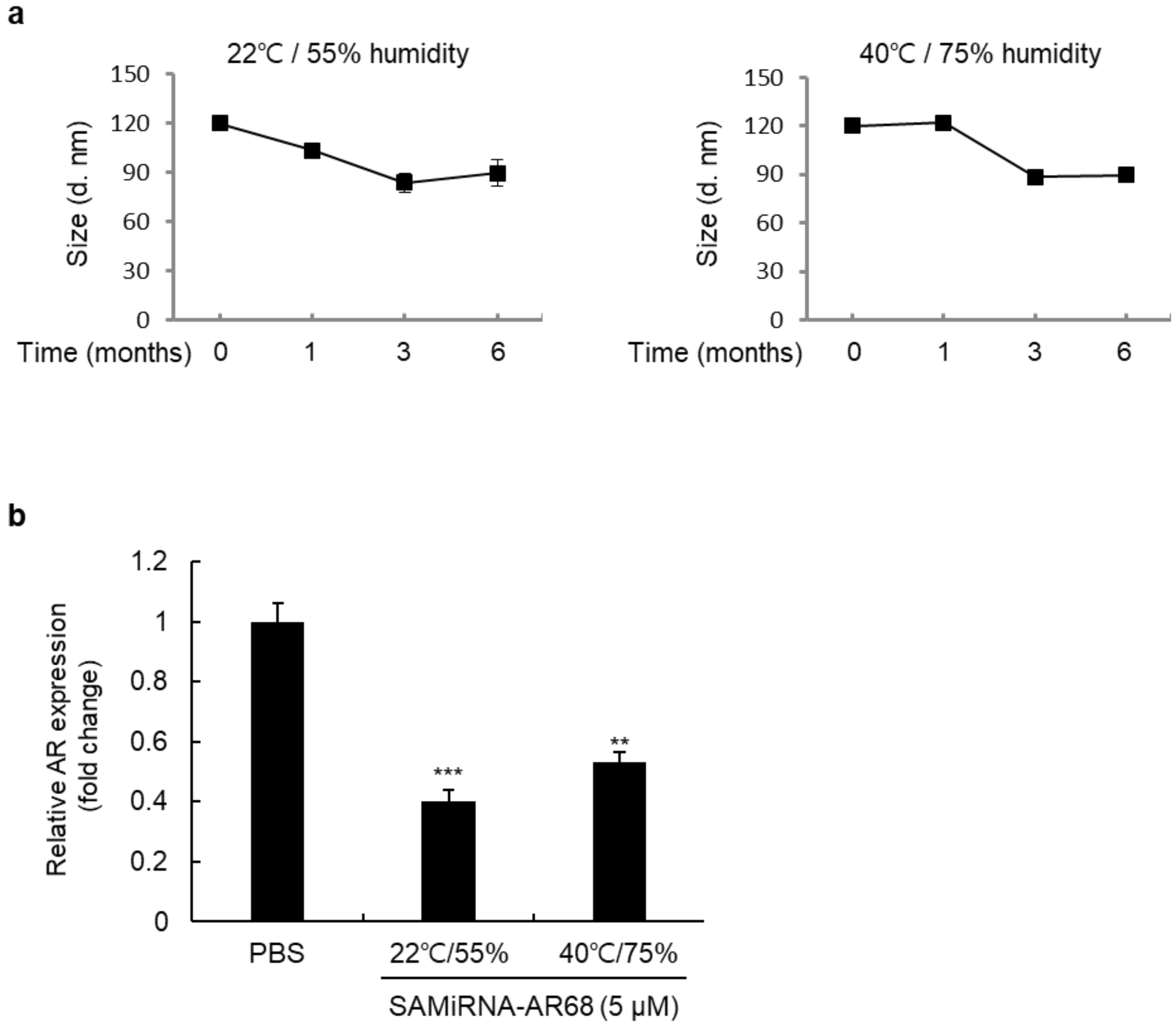

Supplementary Figure S2. The stability of SAMiRNA-AR68. (a) The sizes of AR68 nanoparticles were measured by a qNano gold to assess the long-term stability of SAMiRNA over 6 months. (b) HFDP cells were treated with PBS or indicated conditions of SAMiRNA-AR68 (5 μM) for 24 hr. Total RNA extracts were subjected to qPCR assays, and AR expression was normalized to that of the RPLP0 gene.

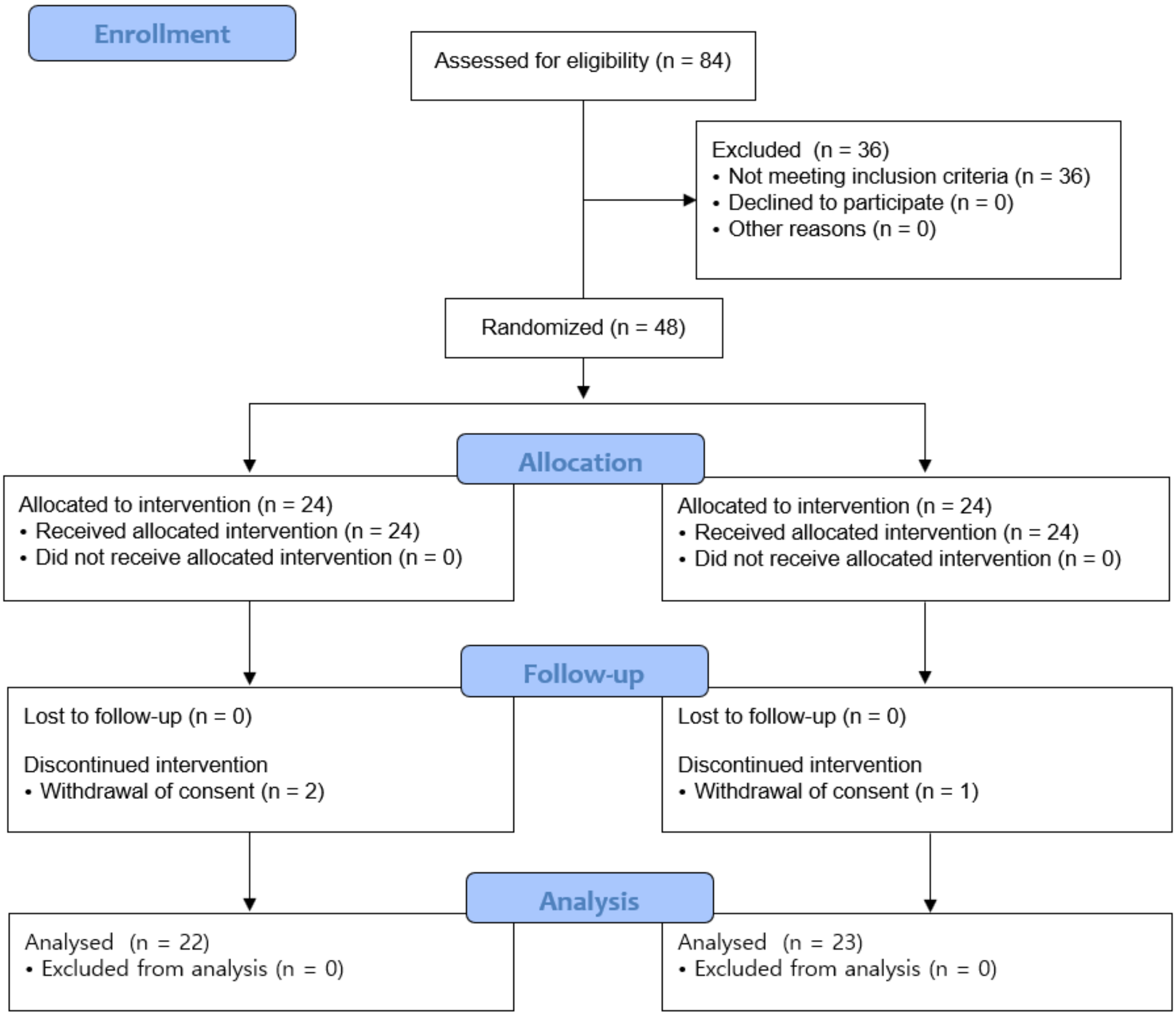

Supplementary Figure S3. Flow diagram of clinical study I. Diagram showing the flow of subjects in the study of SAMiRNA-AR68(0.5 mg/ml) and placebo.

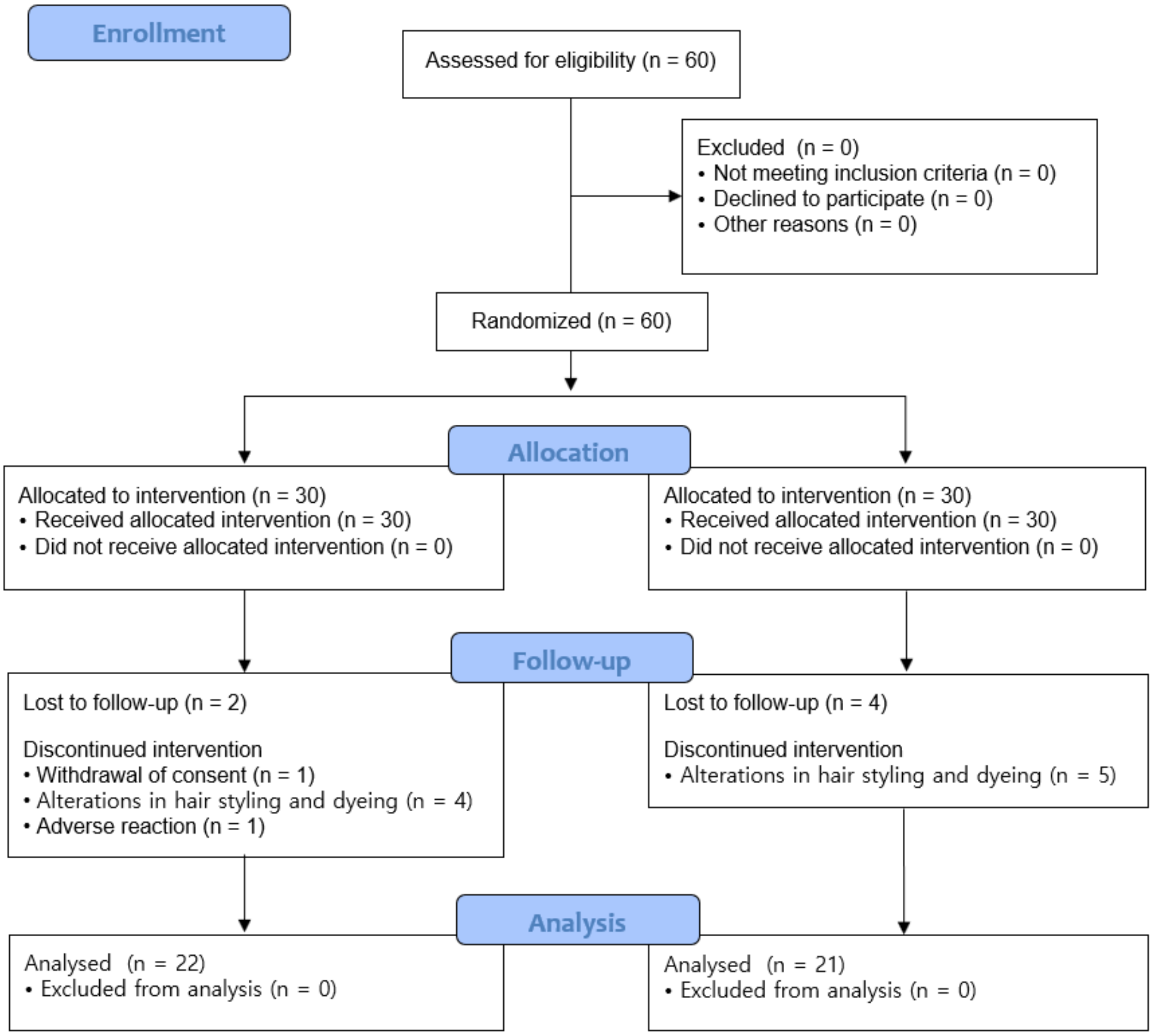

Supplementary Figure S4. Flow diagram of clinical study II. Diagram showing the flow of subjects in the study of SAMiRNA-AR68(5 mg/ml) and placebo.

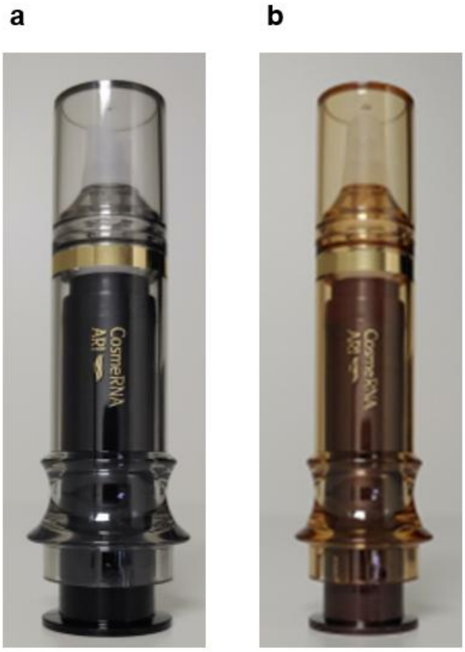

Supplementary Figure S5. The photograph of piston type bottle with silicon adaptor containing SAMiRAN-AR68. Both bottle has same dimension, but the color is different depending on the concentration of SAMiRNA-AR68 (a) 0.5 mg/ml, (b) 5 mg/ml.

Source data for Figure 1c

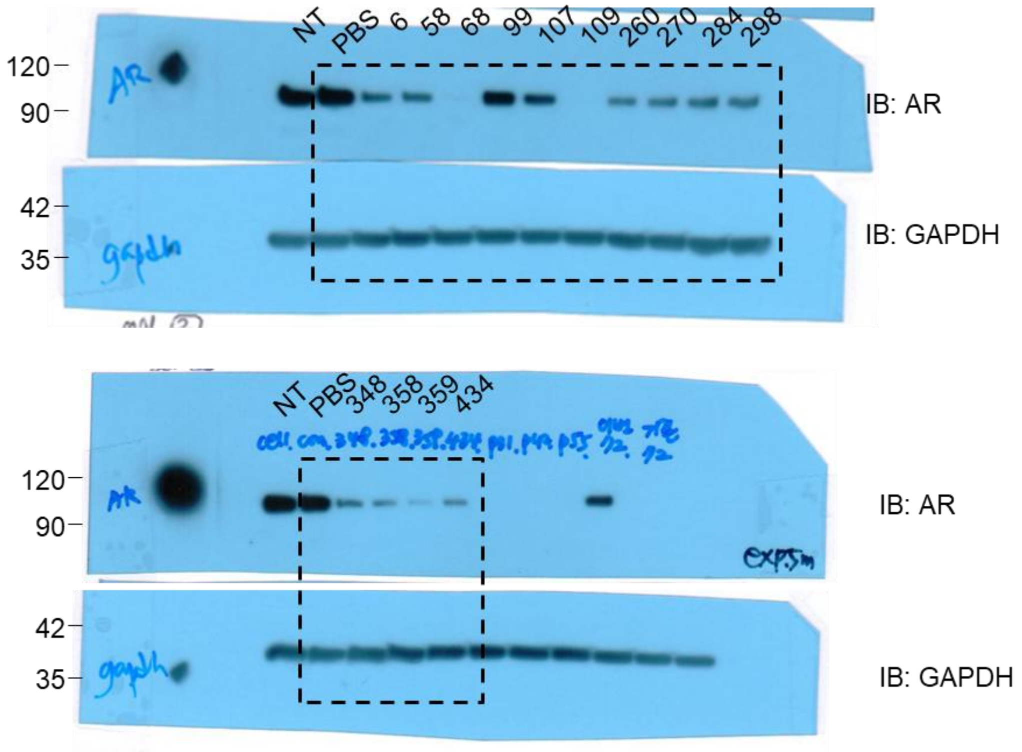

Source data for Figure 2b

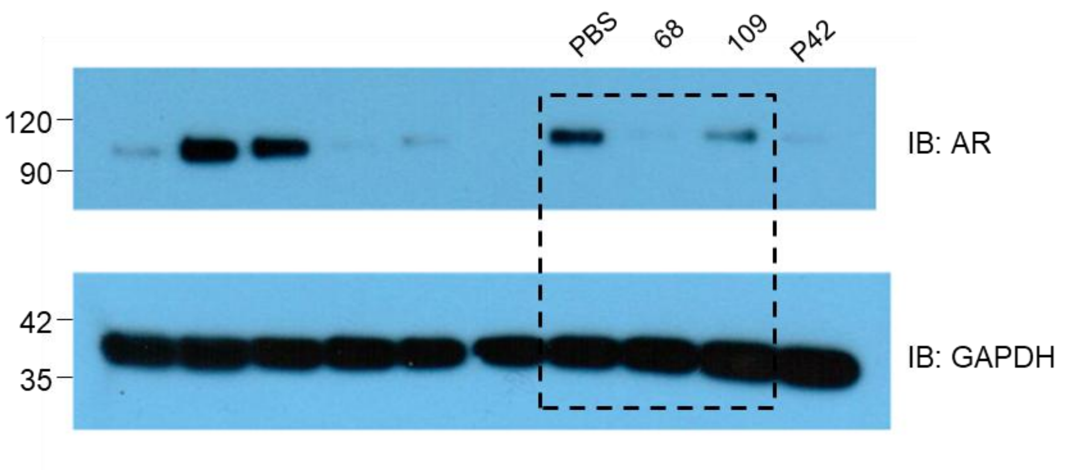

Supplement: Supplementary file 1 — Supplementary Information. [file 41598_2022_5544_MOESM1_ESM.pdf]
